# From raw data to data-analysis for magnetic resonance spectroscopy – the missing link: jMRUI2XML

**DOI:** 10.1186/s12859-015-0796-5

**Published:** 2015-11-09

**Authors:** Victor Mocioiu, Sandra Ortega-Martorell, Iván Olier, Michal Jablonski, Jana Starcukova, Paulo Lisboa, Carles Arús, Margarida Julià-Sapé

**Affiliations:** 1 Departament de Bioquímica i Biologia Molecular, Universitat Autònoma de Barcelona, UAB, Cerdanyola del Vallès, Barcelona, 08193 Spain; 2 Centro de Investigación Biomédica en Red en Bioingeniería, Biomateriales y Nanomedicina CIBER-BBN, Cerdanyola del Vallès, Barcelona, Spain; 3 Institut de Biotecnologia i Biomedicina (IBB), Universitat Autònoma de Barcelona, Cerdanyola del Vallès, Barcelona, Spain; 4 School of Computing and Mathematical Sciences, Liverpool John Moores University, Liverpool, UK; 5 Institute of Biotechnology, The University of Manchester, Manchester, UK; 6 Institute of Scientific Instruments of the CAS, v. v. i, Brno, Czech Republic

**Keywords:** Magnetic resonance spectroscopy, Pattern recognition, Signal processing, Software development

## Abstract

**Background:**

Magnetic resonance spectroscopy provides metabolic information about living tissues in a non-invasive way. However, there are only few multi-centre clinical studies, mostly performed on a single scanner model or data format, as there is no flexible way of documenting and exchanging processed magnetic resonance spectroscopy data in digital format. This is because the DICOM standard for spectroscopy deals with unprocessed data.

This paper proposes a plugin tool developed for jMRUI, namely jMRUI2XML, to tackle the latter limitation. jMRUI is a software tool for magnetic resonance spectroscopy data processing that is widely used in the magnetic resonance spectroscopy community and has evolved into a plugin platform allowing for implementation of novel features.

**Results:**

jMRUI2XML is a Java solution that facilitates common preprocessing of magnetic resonance spectroscopy data across multiple scanners. Its main characteristics are: 1) it automates magnetic resonance spectroscopy preprocessing, and 2) it can be a platform for outputting exchangeable magnetic resonance spectroscopy data. The plugin works with any kind of data that can be opened by jMRUI and outputs in extensible markup language format. Data processing templates can be generated and saved for later use.

The output format opens the way for easy data sharing- due to the documentation of the preprocessing parameters and the intrinsic anonymization - for example for performing pattern recognition analysis on multicentre/multi-manufacturer magnetic resonance spectroscopy data.

**Conclusions:**

jMRUI2XML provides a self-contained and self-descriptive format accounting for the most relevant information needed for exchanging magnetic resonance spectroscopy data in digital form, as well as for automating its processing. This allows for tracking the procedures the data has undergone, which makes the proposed tool especially useful when performing pattern recognition analysis. Moreover, this work constitutes a first proposal for a minimum amount of information that should accompany any magnetic resonance processed spectrum, towards the goal of achieving better transferability of magnetic resonance spectroscopy studies.

**Electronic supplementary material:**

The online version of this article (doi:10.1186/s12859-015-0796-5) contains supplementary material, which is available to authorized users.

## Background

Magnetic Resonance Spectroscopy (MRS) is the twin sister of the more popular Magnetic Resonance Imaging (MRI). MRS is a non-invasive measure of the chemical composition of the investigated volume, also known as voxel. MRS can be Single Voxel (SV) where the signal originates from a single volume, or Multi Voxel (MV) where the signals are read from multiple adjacent voxels, allowing to create metabolic maps or nosological images [1]. After acquisition, MRS data needs to undergo preprocessing steps, such as residual water peak removal in proton MRS (^1^H-MRS) [2]. Even though some common processing parameters are frequently applied, their exact parameter values can vary depending on the particular processing protocol agreed for the study. After processing, spectra are saved in a defined data format, usually specific to the software program used for the processing. Then, data can be input into data-analysis software to answer a relevant question; for example to gain insights for a more accurate pre-operative orientation during a radiological evaluation of an abnormal brain mass [3].

Multicentre studies with in-vivo MRS have traditionally been performed either on a single scanner model or on scanners of the same manufacturer [4–6]. This eliminates the need for unified output format for subsequent data analysis, but at the same time restricts the number of cases that can be included in the study. The latter is especially relevant when undertaking multicentre studies of diseases with very low prevalence, such as some classes of brain tumours [7]. Only a few multicentre studies [8–10] involving the classification of brain tumours using MRS data, have included different scanner manufacturers. These studies acquired raw spectra with variable resolutions, i.e. points/ppm, where ppm accounts for the spectral frequency range, due to variability in manufacturer formats and slightly different acquisition conditions. Because the intended analysis type was pattern recognition, it was necessary to devise a common output format that would, among other characteristics, ensure the same number of spectral points in the same spectral frequency range across all manufacturers. This was successfully achieved during the INTERPRET project with the creation of a canonical format that used 512 points to represent the range between 7.1 to −2.7 ppm. It was a simple ASCII text file with numbers separated by spaces, where the first point corresponds to the spectral intensity at 7.1 ppm [10, 11]. The problem was that this format has no accompanying information and the automated software that generated it could only deal with SV spectra from formats available up to 2002. Later on, two other multicentre projects, eTumour [9] and HealthAgents [8], adopted the INTERPRET processing software, but during the period 2004–2009 the increase in the variety of formats from the main scanner manufacturers was already unmanageable [9], and a bypass through jMRUI [12] was devised as a standalone converter from jMRUI text output to the INTERPRET canonical format [11].

jMRUI [12] is one of the most popular programs for MRS data processing, together with LCmodel [13]. Essentially, the standalone converter mentioned before took the jMRUI output and interpolated (or truncated) to obtain the same number of points across different manufacturers. These projects also had a practical aspect that required a common processing pipeline and output format: the huge number of spectra gathered – in the order of the thousands- as well as the requirements for automated quality control and display in the respective databases, which made manual processing an impractical task. For SV, the jMRUI bypass had the drawback that *de facto* it could never ensure that the spectroscopist had followed exactly the same processing pipeline. For MV, the common output format was simply non-existent and never developed; therefore quality control had to rely on screenshots of the processed files by the scanner’s console. Hence there are some gaps that need to be solved for future multicentre studies using MRS: there is a need for a self-contained, manufacturer-independent format for processed SV and MV data.

In the magnetic resonance field, the Digital Imaging and Communication in Medicine (DICOM) [14] format is routinely used, however this format deals with raw time domain data (unprocessed), despite that there have been efforts to output processed files in DICOM [15].

We have developed a preprocessing tool in the form of a jMRUI plugin that benefits from the capabilities of jMRUI in terms of its capacity to read almost any MRS format, as well as the variety of processing methods available. We have added the necessary implementation based on the eXtensible Markup Language (XML) [16] to export the preprocessed MRS as well as the preprocessing parameters as metadata.

## Implementation

jMRUI2XML has a graphical user interface (GUI) developed in Java on the jMRUI plug-in platform, and adheres to the Model View Controller design pattern. It is aimed at two goals, firstly, automating MRS processing, and secondly, serving as a platform for outputting exchangeable data for use in pattern recognition. jMRUI’s current architecture allows for easy development of four categories of plugins (conversion, preprocessing, quantitation, and custom) via interface implementation [12].

**Fig. 1:**
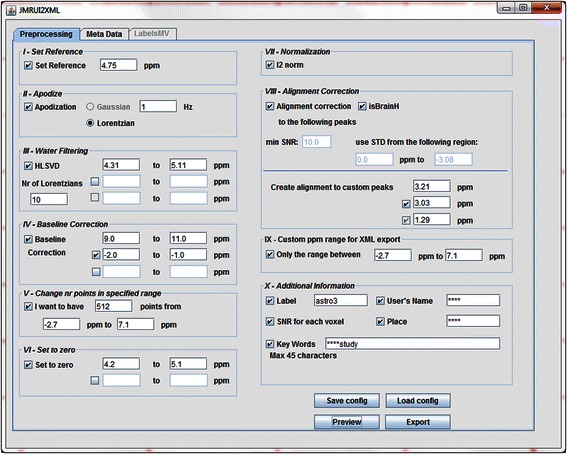
The jMRUI2XML interface with the Pre-processing tab in foreground. The users may select which steps to apply to the spectrum by ticking the ones they are interested in. The steps are performed in the order of their roman numbering –the lower numbers get performed first

The jMRUI2XML GUI is aimed at being a user-friendly and straightforward tool; resembling a form, where the users select only the steps they are interested in. It consists of three tabs: Preprocessing, Meta Data and LabelsMV (see Fig. 1). The first tab contains all the Preprocessing steps along with some additional information that can be added to the output. The second tab contains a text-editor-like interface that is also added to the output, and the last tab will be used in case the user wants to provide labels for the individual voxels of a MV acquisition. jMRUI2XML saves the processed spectra along with preprocessing details in an XML file.

### jMRUI2XML functionality and interface description

#### Pre-processing tab

Fig. 1 shows the interface with the *Preprocessing* tab in foreground. This tab entails ten steps labelled with a roman number and a descriptive name. The first eight steps (I through VIII) are purely preprocessing steps and the last two (IX and X) serve as meta-data input. It must be said that the preprocessing steps are performed in increasing order of their roman numbering – the lower numbers are performed first.

The first three steps, i.e. *Set Reference*, *Apodize*, and *Water filtering,* were already available algorithms in jMRUI*.* Step I, *Set Reference*, takes as input a value in ppm that will represent the new reference. For example, one may set the reference to 4.75 ppm for unsuppressed water in proton spectra. Step II, *Apodize*, is used for line broadening with either a Gaussian or a Lorentzian function of a certain line-width (input given in Hz). Step III, *Water Filtering*, uses the HLSVD algorithm [17, 18] to commonly filter the water signal in case of proton spectra. The interface requires as input the number of Lorentzians to be used and can filter simultaneously up to three regions (units for filtered range in ppm). By allowing multiple filtering regions the users can filter any signal they need, not only water. We emphasize this because our plugin should not be seen as being limited to proton spectra. Step II and III are carried out in the time-domain.

In step IV, *Baseline Correction*, users may choose up to three regions (units for range to correct in ppm) to use in performing baseline correction. The means of the data points heights in the given ranges are summed up and their mean is taken; this value is then subtracted from each data point height of the spectral vector. The algorithm can only perform appropriate correction to a baseline that is not sloped.

Step V, *Change number of points in specified range*, allows the user to enforce a strict number of points in a certain ppm range. If the spectra have more points in the specified range then the algorithm will undersample the frequency vector by skipping values; if there are less points than desired then the algorithm does zero filling (in the time domain), as required. This is the step that allows the user to homogenize the spectral range and number of points, for example two spectra, one with spectral width of 2500 Hz and 2048 points and the other with 1000 Hz and 512 points.

*Set to zero*, the VI-th step, can set to 0 the spectral points in up to two intervals (in ppm). This step can be useful, for example in ^1^H-MRS, when water suppression was poorly performed yet the user needs that residual contribution, for example to vector normalization, to be negligible.

Step VII – Normalization will perform l^2^ normalization [19], also named Unit Length, on the spectra. Currently only this type of normalization is defined in the plugin. The definition of an l^2^ norm of is shown in Eq. 1:

1 $$ \left|x\right| = \sqrt{{\displaystyle \sum_{k=1}^n}\left|{x}_k\right|{}^2} $$

where *n* is the number of spectral points in the spectral vector *x*. An l^2^ normalized spectrum will have each value from k = 1 to n divided by the l^2^ norm, |x|.

It is recommended to perform this step because the amplitude of the spectrum is expressed as arbitrary units and may vary from scanner to scanner. The l^2^ normalization brings all spectra within the same relative range on the y axis. Again, if the purpose is to unify the number of points and the sweep widths that step (referring to step V) must be performed before Step VII.

Finally, the last procedure that can be applied is Step VIII, *Alignment Correction*. This eighth step will correct for slight misalignments of the spectrum according to a theoretical value of some preset reference peak(s) (±10 points in the frequency vector – in the frequency domain, note that this means a smaller ppm range for higher resolution data). If one chooses this step, the first thing is to let the system know if the spectra to be aligned are brain proton spectra. If they are and the *isBrainH* checkbox is ticked then all relevant fields are automatically filled in and the alignment algorithm for 1.5 T SV spectra described in [11] is used. The second option is to input manually the theoretical values for the peaks that will be used for alignment. Then, the algorithm aligns according to the peak with the highest signal-to-noise ratio (SNR) amongst the previously defined ones. The SNR is computed using Eq. 2:

2 $$ SNR = \frac{Max\  Peak}{2STD(noise)} $$

where *Max Peak* represents the highest amplitude in the spectral vector, *STD* stands for standard deviation and in this particular case we consider the *noise* from a range chosen by the user.

The above preprocessing steps (from V onwards) were not available beforehand in jMRUI and they have been adapted from the INTERPRET data manipulation software [10, 11]. With the exception of zero filling, the new steps act in the frequency domain in contrast to the way jMRUI works (in the time domain).

Steps IX and X do not directly affect the spectrum or spectra present in jMRUI. On the contrary, they deal with exporting details - for example, even though the user may choose in the fifth (*Change number of points in specified range*) step to have a specific number of points in a given ppm interval it does not mean that the plugin will output only that range (unless otherwise instructed the plugin will output the full initial ppm range, while ensuring the desired number of points in the selected ppm range). This step, IX, is used when the user only needs a specific ppm range to be present in the output. This is useful when dealing with data from multiple machines which have different ppm ranges before the processing.

For example, when dealing with two raw spectra that have the same number of points but slightly different ppm ranges this step is needed because otherwise outputting the full ppm range/spectra will result in spectral vectors with different number of points.

Moving forward to the *Additional Information* step (X), it is compulsory to complete the *User’s Name* and *Place* fields if one wants to be able to export the processed spectrum as an XML file. This was done because it ensures a minimum traceability on who (and where) has performed the preprocessing. The date of the processing is automatically assigned by the plugin when exporting to XML.

Other information that can be written to the XML is: the SNR for each voxel (which uses Eq. (2), with the highest peak in the whole spectrum as Max Peak), the class label in case of single voxel. By default, the ‘***’ value will be given to the label tag; otherwise the user can label the spectrum as “tumour”, “normal”, etc.

Keywords can also be added with a maximum of 45 characters. This is relevant when the user wants to retain pin-pointed details about the spectra, for example “treated with substance X” or “acquired X minutes after substance injection”.

**Fig. 2:**
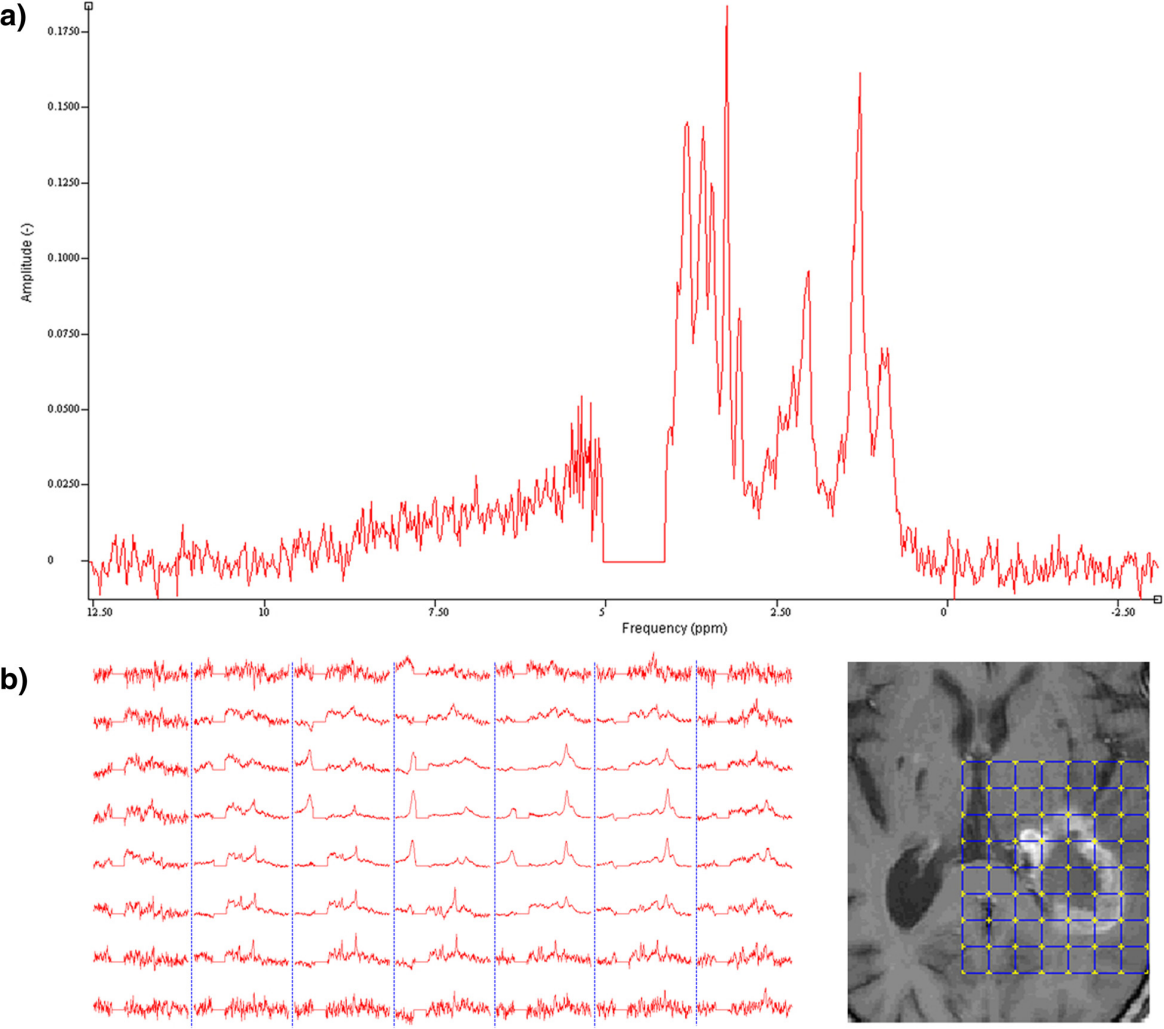
Spectra preprocessed using jMRUI2XML (visualized in jMRUI). **a** An example of a SV spectrum that underwent all the possible steps provided in the jMRUI2XML pipeline (see Fig. 1 for parameters used). **b** The spectral MV grid (left) and the reference image showing where the MV was recorded (right). The MV grid has undergone the same steps ([11]) as in **a**)

Finally the four control buttons serve as follows: *Save config.* saves the parameters that are present in the interface at button-press time to an XML file that can later be loaded back using *Load config. Preview/Original* can be toggled to see how spectra will look like by tuning parameters and once the user is satisfied the spectra can be saved as an XML file by using the *Export* button. Such a processed spectrum can be seen in Fig. 2.

It is useful to note that the jMRUI2XML plugin uses the data that is available when it is loaded; if the spectrum has been processed before loading it, then the plugin will see that spectrum and not the unprocessed one. The same is valid if the users apply other processing steps after they have clicked *Preview* but before *Export*, for example if they apply additional zero or first order phasing manually using the jMRUI main menu.

### Meta Data tab

**Fig. 3:**
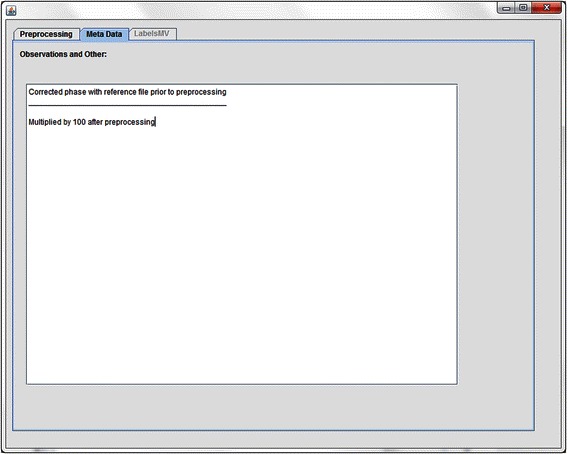
The Meta Data tab. Example of usage where the user wants to store that the spectrum was manually phased and multiplied by a scalar (100) available through the jMRUI main menu, after going through the jMRUI2XMLs preprocessing pipeline

The second tab- *Meta Data* (Fig. 3) serves as an observation notebook. For instance, as in the previous example when manual phasing or any extra procedure available in jMRUI but not in the plugin has been applied through its main menu, this is where it can be manually recorded using this tab. Although it might seem trivial, this tab can accommodate details that are crucial for later data-analysis, such as voxel position, clinical trial code, comments about spectral quality or even a description of the spectrum in report terms.

### Labels MV tab

**Fig. 4:**
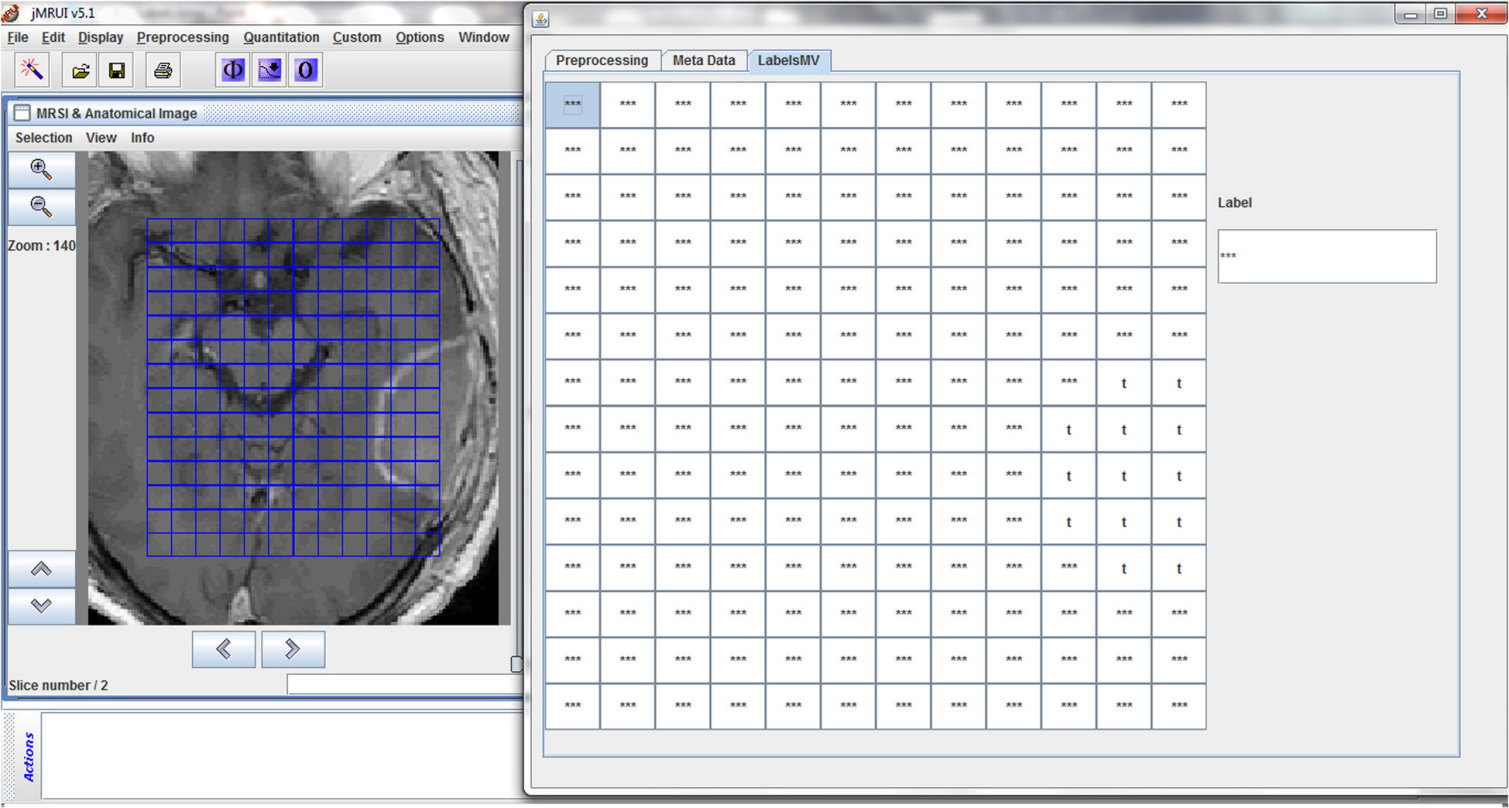
The LabelsMV tab. It is in this tab where each individual voxel can be assigned the desired label. In this case please note that the voxels that are overlaid to the abnormal tissue have been labelled by the user with “t” whereas the rest are left with the default label “***”

The last tab of jMRUI2XML is called LabelsMV and it is used for labelling each individual voxel of an MV grid. Fig. 4 depicts this tab - each cell in the grid represents a voxel; each of them has a label assigned with the default value of “***”. The labels can be viewed and edited by clicking on a cell. The grid follows matrix notation, meaning the top left corner corresponds to position [1, 1] and the bottom right corner corresponds to position [m,n], where m is the number of rows and n is the number of columns.

## Results

### Standardized XML schema definition for data exchange

**Fig. 5:**
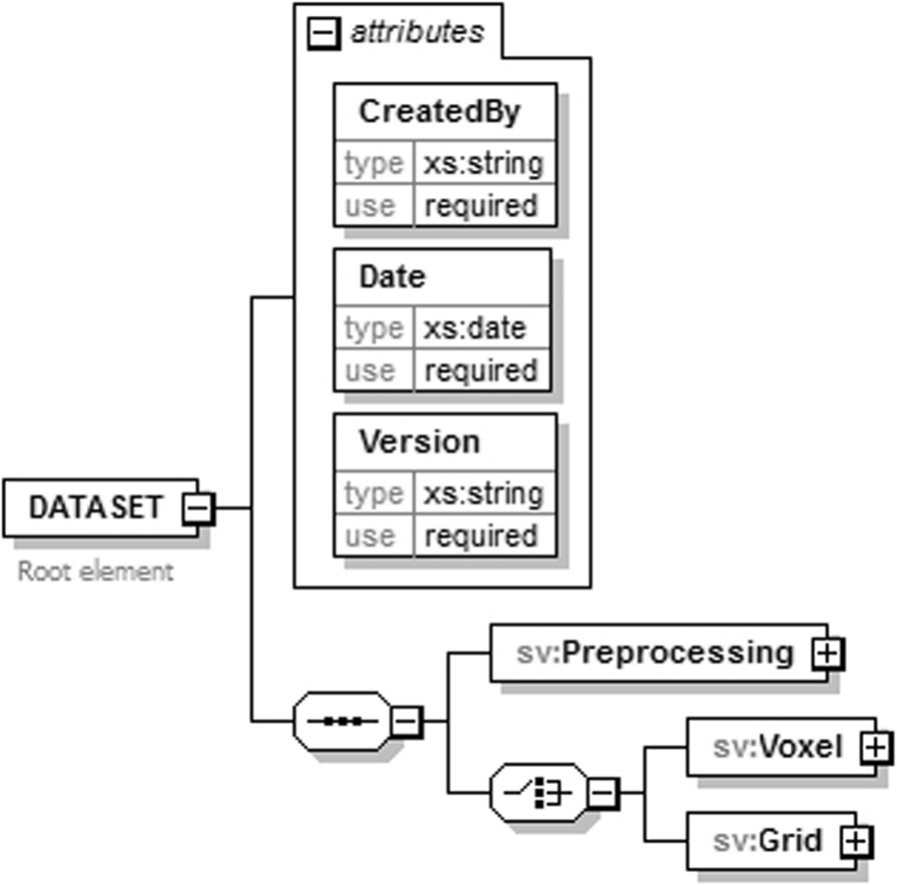
Structure of the DATASET node. The root node, DATASET, has two children: the Preprocessing node and either a Voxel node or a Grid node. The Preprocessing node will encompass the preprocessing history that was performed using jMRUI2XML. The Voxel node contains the actual data, and the Grid node may contain multiple Voxel nodes

**Fig. 6:**
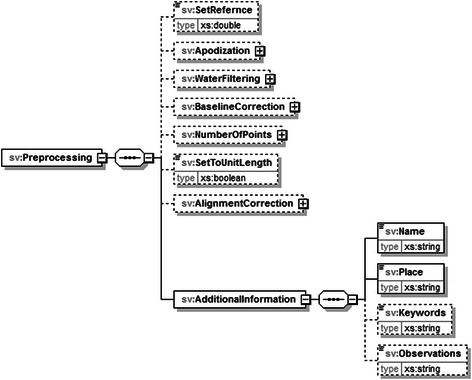
Structure of the Preprocessing node. This node may contain all the preprocessing steps that were performed using jMRUI2XML. The dashed lines mean that a node is optional; absence of a node indicates that the corresponding step was not performed by jMRUI2XML. Note that only the *AdditionalInformation* node is always required along with the *Name* and *Place* sub-nodes

**Fig. 7:**
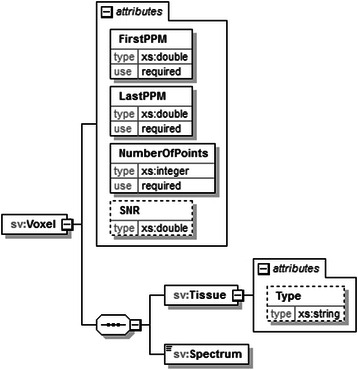
Structure of the Voxel node. In case of a SV this node holds the preprocessed data, along with various details about it

**Fig. 8:**
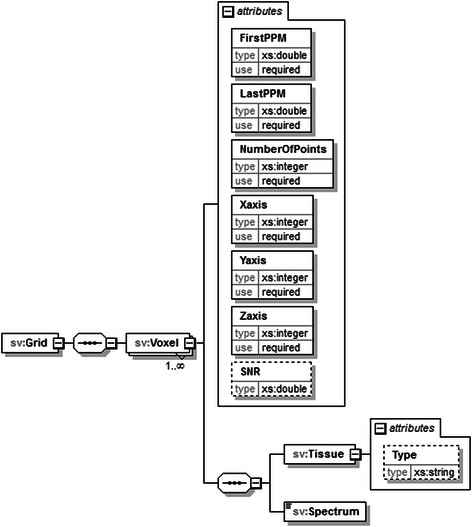
Structure of a Grid node. This node will hold MV data and it comprises of multiple Voxel nodes similar to the previous but with three extra attributes. These are meant to account for a voxel’s position in the grid

jMRUI2XML’s XML output, to which we will hereafter refer to as format, draws its inspiration from the schema proposed in [20]. Similarly we use DATASET as the root node for a file, but we only accommodate one case (either SV or MV) per file (Fig. 5). Another novelty with respect to the XML schema defined in [20] is that we retain the preprocessing steps that were undertaken using jMRUI2XML, and also allow for other details to be saved (Fig. 6). In this iteration of the schema we consider the main data node to be either *Voxel* (Fig. 7 ) for SV or *Grid* (Fig. 8) for MV.

One thing to note is that the Voxel sub-node of the Grid has three attributes, Xaxis, Yaxis and Zaxis that can be used to retain a voxel position within the grid; however because jMRUI works with one slice at any given time the Zaxis attribute will always be 1. Nevertheless, we have included this in the format anticipating a future possibility of manipulating multiple slices simultaneously. For further information on the exchange format please refer to the additional materials. The distribution of the plugin also includes an XML template for the INTERPRET canonical format and two scripts – one for MATLAB and one for python. The MATLAB script reads in the above format as a structure (where the spectral data is either a 1D vector or a 2D array) whereas the python reads it in as a dictionary (where the spectral data is a numpy array).

### Evaluation

We have evaluated the speed of the plugin by using a proton 16 by 16 MV grid with 1024 points per raw spectrum. We used the INTERPRET parameters for preprocesing, set some keywords (Short TE, 1.5 T), entered 5 paragraphs of lorem ipsum [21] in the Meta Data tab and randomly labelled 50 % of the voxels. It takes ~22 seconds from button press to XML exportation on a core i7@2.6 GHz; memory consumption is around 120 MB RAM. We ran the same test also with a 10 by 12 MV grid with 512 points per raw spectrum and one high-resolution SV spectrum with 8192 points on which the processing took ~7 seconds and ~ 1 second respectively. The voxels are processed sequentially so in theory the run time should be linearly dependent (mainly) on the number of voxels.

## Discussion and conclusions

jMRUI2XML is a flexible tool that can represent a wide range of MRS preprocessing procedures. It applies to MRS data from any scanner that can be opened with jMRUI, and its internal structure guarantees that it will always be able to do so. Furthermore, it provides a flexible output format that can hold in the data preprocessing parameters, thus enabling the user to always keep track of what procedures the data has undergone.

Other software for MRS such as Tarquin [22], SIVIC [23] or LCModel [13] provide preprocessing algorithms, however the output is not normally compatible among different acquisition conditions. Tarquin outputs in CSV, and SIVIC in DICOM, but they do not entail preprocessing history. The absence of several control parameters, such as number of points to output, what range to output, etc., make these tools cumbersome to work with when using multi-centre/multi-manufacturer data. If pattern recognition analysis is to be performed on the data then, it is often the case that the same number of points needs to be ensured for a certain ppm range across all data, and it is not always possible to track whether the preprocessing techniques used allow for compatible data. In addition, now it is possible to export processed MV data with the same parameters as for SV, for example the INTERPRET pipeline can be applied now to MV.

jMRUI can output data in their own binary or text format; additionally these can be accompanied with an extra batch file that contains the preprocessing steps. Likewise LCModel can output the data and the preprocessing details in separate files. However, besides the added algorithms and meta-data, our solution keeps all details in one self-contained and self-descriptive file.

Previous endeavors have been put into the usage of XML in the MRS field [20, 24]. For example, the SpectraClassifier (SC) software [20] for performing pattern recognition on MRS data. As such, an XML containing the entered data was generated after the processed MR spectrum in jMRUI format or other formats had been uploaded into the application.

In [24] the authors developed a generalized classification framework in which classifier representation was done via three XML files that worked in conjunction to describe the input data type, such as the question it answered, feature selection, performance metrics, etc. However the input data was assumed to be ASCII files adhering to a particular canonical format [10], therefore ignoring the preprocessing step and how to document it for further data exchange. This means that any spectrum with the correct number of points (i.e. 512) will do fine as input, regardless of whether it went through the same preprocessing steps or not. This limits the extent to which any automatic system can guarantee for the robustness of a classifier regardless of apparent performance on a training set.

Indeed, from a purely pattern recognition point of view one would only need the data vector, however presence or absence of the preprocessing details complementing the spectral data enables various distinct usage scenarios. For example, a homogeneous, traceable data processing pipeline allows for decentralized data processing. This is relevant in clinical trials where a common format can serve quality control and quality assurance for documenting withdrawals. A not so obvious property of jMRUI2XML is anonymization; this is an intrinsic property of the output which can allow data sharing among research centres. In multi-centre studies this provides the technical framework for sharing de-identified processed data for sending to one or multiple data analysis centres.

jMRUI2XML has immediate applicability when combined with the newly released version of SC as a jMRUI plugin [25] or the INTERPRET decision-support system [26] (DSS), also a jMRUI plugin. When placed in the appropriate folder, SC reads the XML files automatically which can then be used to build various classifiers; and the INTERPRET DSS can be used with SV data for decision support. In this way, the jMRUI2XML integrates data processing with classification and decision-support, working as plugins of the jMRUI processing software.

Regarding possible improvements, in future versions we hope for a tighter integration with the jMRUI core. For example, preprocessing steps or manipulations applied to the data, which are not available within the plugin, could be automatically added to the Meta Data tab. Another possible improvement would be to coregister the current LabelsMV tab with the reference MR image.

Our work also constitutes a first proposal for a minimum amount of information that should accompany any MR processed spectrum, towards the goal of achieving better transferability of MRS studies. The concept of “minimum information about an experiment” has been put forward and is already in use by different communities of biological researchers [27]. The MIAME standard, “Minimum information about a microarray experiment” as well as the MAGE-TAB “Microarray Gene Expression Markup Language”, are the most known and successful examples [28, 29]. MIAME builds upon two principles: 1) the information about the experiment should be sufficient to interpret it and allow comparisons with similar experiments as well as permit replication of that experiment, and 2) the information should be structured in a way that enables useful querying as well as automated data analysis and mining. In this sense, our proposed output XML schema Additional file 1 should fulfill both principles and constitute a first step towards a future definition of the “Minimum information about an MRS experiment”. Along the same lines, the MR community has already developed a set of guidelines for fMRI data [30], but a similar initiative is still lacking in the MRS arena despite recent community initiatives to develop guidelines for data acquisition and analysis, quality assessment, and interpretation [31].

An ongoing project, COSMOS (Coordination of Standards in Metabolomics) is currently developing nmrML [32], an open mark-up language for MRS data. However at the moment it is limited to metabolomics, high-resolution NMR data. This format deals mainly with acquisition parameters, and although a proposal for a preprocessing node exists in their specifications it has yet to be implemented in their XML schema [33].

In conclusion, jMRUI2XML is a software tool that extends jMRUI's processing power with new algorithms, and provides a flexible preprocessing framework accompanied by a versatile output format allowing researchers to exchange data and to focus on analysis rather than on data preprocessing.

### Availability and requirements

Project name: *jMRUI2XML*

Project home page: http://gabrmn.uab.es/jmrui2xml

Operating system(s): Platform independent

Programming language: Java

Other requirements: jMRUI

License: Available free of charge

Any restriction to use by non-academics: Subject to the signature of a disclaimer and user agreement text available at the project homepage.

## Additional file

Additional file 1:
**XML Schema Definition.** (PDF 358 kb)

## Abbreviations

COSMOS: Coordination of Standards in Metabolomics
DICOM: Digital Imaging and Communication in Medicine
MRS: Magnetic resonance spectroscopy
MV: Multi-Voxel
PR: Pattern recognition
SV: Single-Voxel
TE: Echo time
XML: Extensible markup language
