# Supplementary material for: From raw data to data-analysis for magnetic resonance spectroscopy – the missing link: jMRUI2XML
Source: BMC Bioinformatics. 2015 Nov 9;16:378. doi: 10.1186/s12859-015-0796-5 (PMC4640235; doi:10.1186/s12859-015-0796-5)
Supplement: Additional file 1: — XML Schema Definition. (PDF 358 kb) [file 12859_2015_796_MOESM1_ESM.pdf]

Elements
DATASET

element **DATASET**

diagram

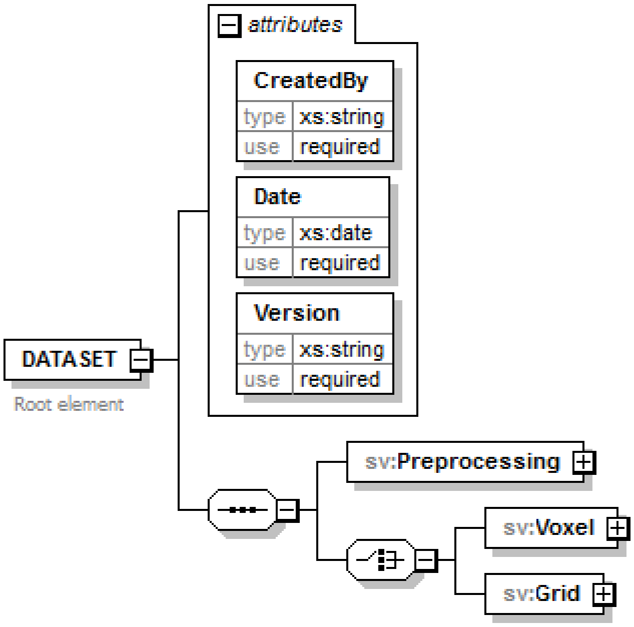

namespace	http://gabrmn.uab.es/jmrui2xml_sv
properties	content complex
children	**sv:Preprocessing** **sv:Voxel** **sv:Grid**

attributes

Name	Type	Use	Default	Fixed	Annotation
CreatedBy	**xs:string**	required			
Date	**xs:date**	required			
Version	**xs:string**	required			

annotation

documentation
Root element

source

```
<xs:element name="DATASET">
 <xs:annotation>
  <xs:documentation>Root element</xs:documentation>
 </xs:annotation>
 <xs:complexType>
  <xs:sequence>
   <xs:element name="Preprocessing">
    <xs:complexType>
     <xs:sequence>
      <xs:element name="SetRefernce" type="xs:double" minOccurs="0"/>
      <xs:element name="Apodization" minOccurs="0">
       <xs:complexType>
        <xs:choice>
         <xs:element name="Lorentzian">
          <xs:complexType>
           <xs:sequence>
```

	(continued below)

```xml
            <xs:element name="Hz" type="xs:integer"/>
          </xs:sequence>
        </xs:complexType>
      </xs:element>
      <xs:element name="Gaussian">
        <xs:complexType>
          <xs:sequence>
            <xs:element name="Hz" type="xs:integer"/>
          </xs:sequence>
        </xs:complexType>
      </xs:element>
    </xs:choice>
  </xs:complexType>
</xs:element>
<xs:element name="WaterFiltering" minOccurs="0">
  <xs:complexType>
    <xs:sequence>
      <xs:element name="NumberOfLorentzians" type="xs:integer"/>
      <xs:sequence maxOccurs="3">
        <xs:element name="MinPPM" type="xs:double"/>
        <xs:element name="MaxPPM" type="xs:double"/>
      </xs:sequence>
    </xs:sequence>
  </xs:complexType>
</xs:element>
<xs:element name="BaselineCorrection" minOccurs="0">
  <xs:complexType>
    <xs:sequence maxOccurs="3">
      <xs:element name="MinPPM" type="xs:double"/>
      <xs:element name="MaxPPM" type="xs:double"/>
    </xs:sequence>
  </xs:complexType>
</xs:element>
<xs:element name="NumberOfPoints" minOccurs="0">
  <xs:complexType>
    <xs:sequence>
      <xs:element name="NrPoints" type="xs:integer"/>
      <xs:element name="MinPPM" type="xs:double"/>
      <xs:element name="MaxPPM" type="xs:double"/>
    </xs:sequence>
  </xs:complexType>
</xs:element>
<xs:element name="SetToUnitLength" type="xs:boolean" minOccurs="0"/>
<xs:element name="AlignmentCorrection" minOccurs="0">
  <xs:complexType>
    <xs:sequence>
      <xs:element name="isBrainH" type="xs:boolean"/>
      <xs:element name="minSNR" type="xs:double"/>
      <xs:element name="minSTD" type="xs:double"/>
      <xs:element name="maxSTD" type="xs:double"/>
      <xs:element name="firstPeak" type="xs:double"/>
      <xs:element name="secondPeak" type="xs:double" minOccurs="0"/>
      <xs:element name="thirdPeak" type="xs:double" minOccurs="0"/>
    </xs:sequence>
```

```
    </xs:complexType>
   </xs:element>
   <xs:element name="AdditionalInformation">
    <xs:complexType>
     <xs:sequence>
      <xs:element name="Name" type="xs:string"/>
      <xs:element name="Place" type="xs:string"/>
      <xs:element name="Keywords" type="xs:string" minOccurs="0"/>
      <xs:element name="Observations" type="xs:string" minOccurs="0"/>
     </xs:sequence>
    </xs:complexType>
   </xs:element>
  </xs:sequence>
 </xs:complexType>
</xs:element>
<xs:choice>
 <xs:element name="Voxel">
  <xs:complexType>
   <xs:sequence>
    <xs:element name="Tissue">
     <xs:complexType>
      <xs:attribute name="Type" type="xs:string"/>
     </xs:complexType>
    </xs:element>
    <xs:element name="Spectrum"/>
   </xs:sequence>
   <xs:attribute name="FirstPPM" type="xs:double" use="required"/>
   <xs:attribute name="LastPPM" type="xs:double" use="required"/>
   <xs:attribute name="NumberOfPoints" type="xs:integer" use="required"/>
   <xs:attribute name="SNR" type="xs:double"/>
  </xs:complexType>
 </xs:element>
 <xs:element name="Grid">
  <xs:complexType>
   <xs:sequence>
    <xs:element name="Voxel" maxOccurs="unbounded">
     <xs:complexType>
      <xs:sequence>
       <xs:element name="Tissue">
        <xs:complexType>
         <xs:attribute name="Type" type="xs:string"/>
        </xs:complexType>
       </xs:element>
       <xs:element name="Spectrum"/>
      </xs:sequence>
      <xs:attribute name="FirstPPM" type="xs:double" use="required"/>
      <xs:attribute name="LastPPM" type="xs:double" use="required"/>
      <xs:attribute name="NumberOfPoints" type="xs:integer" use="required"/>
      <xs:attribute name="Xaxis" type="xs:integer" use="required"/>
      <xs:attribute name="Yaxis" type="xs:integer" use="required"/>
      <xs:attribute name="Zaxis" type="xs:integer" use="required"/>
      <xs:attribute name="SNR" type="xs:double"/>
     </xs:complexType>
    </xs:element>
```

```
          </xs:sequence>
        </xs:complexType>
      </xs:element>
    </xs:choice>
  </xs:sequence>
  <xs:attribute name="CreatedBy" type="xs:string" use="required"/>
  <xs:attribute name="Date" type="xs:date" use="required"/>
  <xs:attribute name="Version" type="xs:string" use="required"/>
 </xs:complexType>
</xs:element>
```

attribute **DATASET/@CreatedBy**

type	**xs:string**
properties	use required
source	<xs:attribute name="CreatedBy" type="xs:string" use="required"/>

attribute **DATASET/@Date**

type	**xs:date**
properties	use required
source	<xs:attribute name="Date" type="xs:date" use="required"/>

attribute **DATASET/@Version**

type	**xs:string**
properties	use required
source	<xs:attribute name="Version" type="xs:string" use="required"/>

element **DATASET/Preprocessing**

diagram

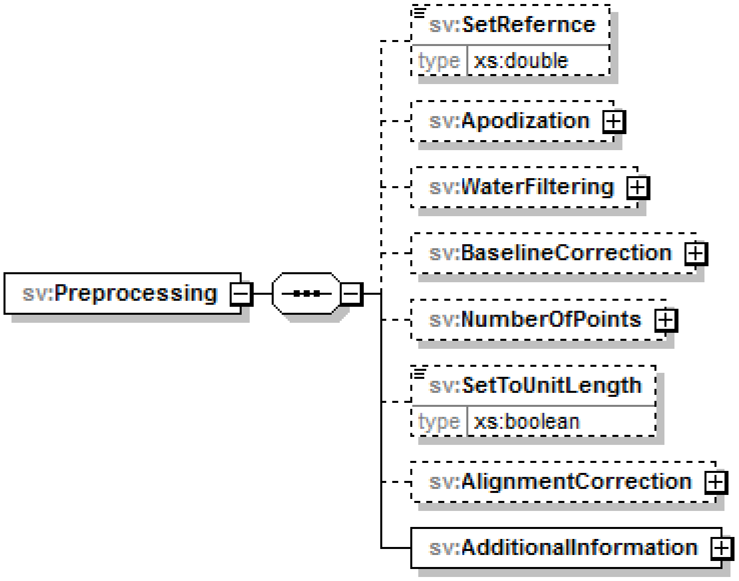

namespace	http://gabrmn.uab.es/jmrui2xml_sv
properties	content complex
children	**sv:SetRefernce** **sv:Apodization** **sv:WaterFiltering** **sv:BaselineCorrection** **sv:NumberOfPoints** **sv:SetToUnitLength** **sv:AlignmentCorrection** **sv:AdditionalInformation**

source

```xml
<xs:element name="Preprocessing">
  <xs:complexType>
    <xs:sequence>
      <xs:element name="SetRefernce" type="xs:double" minOccurs="0"/>
      <xs:element name="Apodization" minOccurs="0">
        <xs:complexType>
          <xs:choice>
            <xs:element name="Lorentzian">
              <xs:complexType>
                <xs:sequence>
                  <xs:element name="Hz" type="xs:integer"/>
                </xs:sequence>
              </xs:complexType>
            </xs:element>
            <xs:element name="Gaussian">
              <xs:complexType>
                <xs:sequence>
                  <xs:element name="Hz" type="xs:integer"/>
                </xs:sequence>
              </xs:complexType>
            </xs:element>
          </xs:choice>
        </xs:complexType>
      </xs:element>
      <xs:element name="WaterFiltering" minOccurs="0">
        <xs:complexType>
          <xs:sequence>
            <xs:element name="NumberOfLorentzians" type="xs:integer"/>
```

```xml
        <xs:sequence maxOccurs="3">
          <xs:element name="MinPPM" type="xs:double"/>
          <xs:element name="MaxPPM" type="xs:double"/>
        </xs:sequence>
      </xs:sequence>
    </xs:complexType>
  </xs:element>
  <xs:element name="BaselineCorrection" minOccurs="0">
    <xs:complexType>
      <xs:sequence maxOccurs="3">
        <xs:element name="MinPPM" type="xs:double"/>
        <xs:element name="MaxPPM" type="xs:double"/>
      </xs:sequence>
    </xs:complexType>
  </xs:element>
  <xs:element name="NumberOfPoints" minOccurs="0">
    <xs:complexType>
      <xs:sequence>
        <xs:element name="NrPoints" type="xs:integer"/>
        <xs:element name="MinPPM" type="xs:double"/>
        <xs:element name="MaxPPM" type="xs:double"/>
      </xs:sequence>
    </xs:complexType>
  </xs:element>
  <xs:element name="SetToUnitLength" type="xs:boolean" minOccurs="0"/>
  <xs:element name="AlignmentCorrection" minOccurs="0">
    <xs:complexType>
      <xs:sequence>
        <xs:element name="isBrainH" type="xs:boolean"/>
        <xs:element name="minSNR" type="xs:double"/>
        <xs:element name="minSTD" type="xs:double"/>
        <xs:element name="maxSTD" type="xs:double"/>
        <xs:element name="firstPeak" type="xs:double"/>
        <xs:element name="secondPeak" type="xs:double" minOccurs="0"/>
        <xs:element name="thirdPeak" type="xs:double" minOccurs="0"/>
      </xs:sequence>
    </xs:complexType>
  </xs:element>
  <xs:element name="AdditionalInformation">
    <xs:complexType>
      <xs:sequence>
        <xs:element name="Name" type="xs:string"/>
        <xs:element name="Place" type="xs:string"/>
        <xs:element name="Keywords" type="xs:string" minOccurs="0"/>
        <xs:element name="Observations" type="xs:string" minOccurs="0"/>
      </xs:sequence>
    </xs:complexType>
  </xs:element>
 </xs:sequence>
</xs:complexType>
</xs:element>
```

element **DATASET/Preprocessing/SetRefernce**

diagram	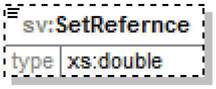
namespace	http://gabrmn.uab.es/jmrui2xml_sv
type	**xs:double**
properties	minOcc 0<br>maxOcc 1<br>content simple
source	`<xs:element name="SetRefernce" type="xs:double" minOccurs="0"/>`

element **DATASET/Preprocessing/Apodization**

diagram	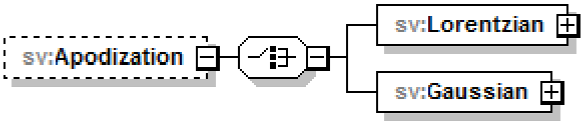
namespace	http://gabrmn.uab.es/jmrui2xml_sv
properties	minOcc 0<br>maxOcc 1<br>content complex
children	**sv:Lorentzian** **sv:Gaussian**
source	see below

```
<xs:element name="Apodization" minOccurs="0">
  <xs:complexType>
    <xs:choice>
      <xs:element name="Lorentzian">
        <xs:complexType>
          <xs:sequence>
            <xs:element name="Hz" type="xs:integer"/>
          </xs:sequence>
        </xs:complexType>
      </xs:element>
      <xs:element name="Gaussian">
        <xs:complexType>
          <xs:sequence>
            <xs:element name="Hz" type="xs:integer"/>
          </xs:sequence>
        </xs:complexType>
      </xs:element>
    </xs:choice>
  </xs:complexType>
</xs:element>
```

element **DATASET/Preprocessing/Apodization/Lorentzian**

diagram	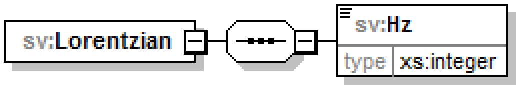

namespace	http://gabrmn.uab.es/jmrui2xml_sv
properties	content complex
children	**sv:Hz**
source	<xs:element name="Lorentzian"><br><xs:complexType><br><xs:sequence><br><xs:element name="Hz" type="xs:integer"/><br></xs:sequence><br></xs:complexType><br></xs:element>

element **DATASET/Preprocessing/Apodization/Lorentzian/Hz**

diagram	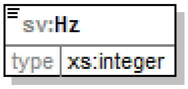
namespace	http://gabrmn.uab.es/jmrui2xml_sv
type	**xs:integer**
properties	content simple
source	<xs:element name="Hz" type="xs:integer"/>

element **DATASET/Preprocessing/Apodization/Gaussian**

diagram	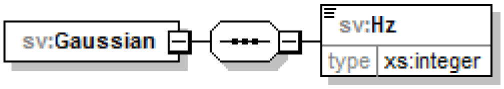
namespace	http://gabrmn.uab.es/jmrui2xml_sv
properties	content complex
children	**sv:Hz**
source	<xs:element name="Gaussian"><br><xs:complexType><br><xs:sequence><br><xs:element name="Hz" type="xs:integer"/><br></xs:sequence><br></xs:complexType><br></xs:element>

element **DATASET/Preprocessing/Apodization/Gaussian/Hz**

diagram	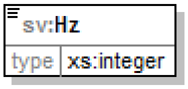
namespace	http://gabrmn.uab.es/jmrui2xml_sv

type	**xs:integer**
properties	content simple
source	<xs:element name="Hz" type="xs:integer"/>

element **DATASET/Preprocessing/WaterFiltering**

diagram	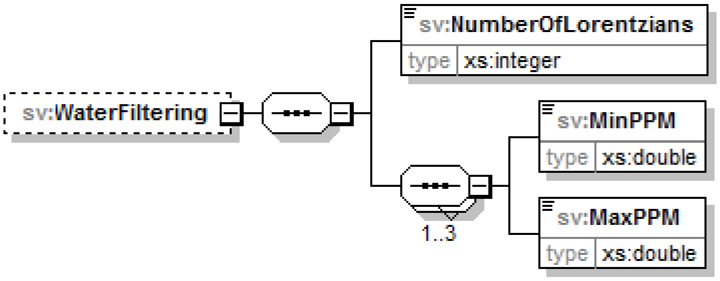
namespace	http://gabrmn.uab.es/jmrui2xml_sv
properties	minOcc 0<br>maxOcc 1<br>content complex
children	**sv:NumberOfLorentzians** **sv:MinPPM** **sv:MaxPPM**
source	<xs:element name="WaterFiltering" minOccurs="0"><br><xs:complexType><br><xs:sequence><br><xs:element name="NumberOfLorentzians" type="xs:integer"/><br><xs:sequence maxOccurs="3"><br><xs:element name="MinPPM" type="xs:double"/><br><xs:element name="MaxPPM" type="xs:double"/><br></xs:sequence><br></xs:sequence><br></xs:complexType><br></xs:element>

element **DATASET/Preprocessing/WaterFiltering/NumberOfLorentzians**

diagram	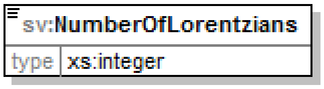
namespace	http://gabrmn.uab.es/jmrui2xml_sv
type	**xs:integer**
properties	content simple
source	<xs:element name="NumberOfLorentzians" type="xs:integer"/>

element **DATASET/Preprocessing/WaterFiltering/MinPPM**

diagram	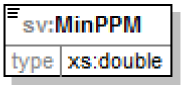
namespace	http://gabrmn.uab.es/jmrui2xml_sv
type	**xs:double**
properties	content simple
source	<xs:element name="MinPPM" type="xs:double"/>

element **DATASET/Preprocessing/WaterFiltering/MaxPPM**

diagram	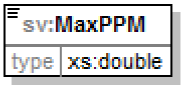
namespace	http://gabrmn.uab.es/jmrui2xml_sv
type	**xs:double**
properties	content simple
source	<xs:element name="MaxPPM" type="xs:double"/>

element **DATASET/Preprocessing/BaselineCorrection**

diagram	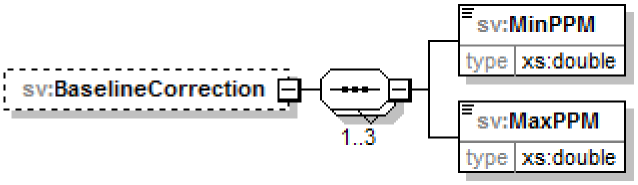
namespace	http://gabrmn.uab.es/jmrui2xml_sv
properties	minOcc 0<br>maxOcc 1<br>content complex
children	**sv:MinPPM** **sv:MaxPPM**
source	<xs:element name="BaselineCorrection" minOccurs="0"><br><xs:complexType><br><xs:sequence maxOccurs="3"><br><xs:element name="MinPPM" type="xs:double"/><br><xs:element name="MaxPPM" type="xs:double"/><br></xs:sequence><br></xs:complexType><br></xs:element>

element **DATASET/Preprocessing/BaselineCorrection/MinPPM**

diagram	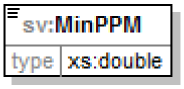
namespace	http://gabrmn.uab.es/jmrui2xml_sv
type	**xs:double**
properties	content simple
source	<xs:element name="MinPPM" type="xs:double"/>

element **DATASET/Preprocessing/BaselineCorrection/MaxPPM**

diagram	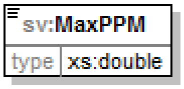
namespace	http://gabrmn.uab.es/jmrui2xml_sv
type	**xs:double**
properties	content simple
source	<xs:element name="MaxPPM" type="xs:double"/>

element **DATASET/Preprocessing/NumberOfPoints**

diagram	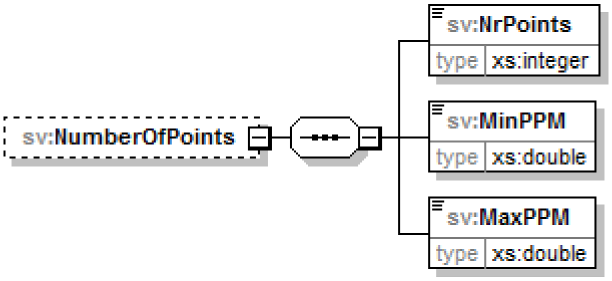
namespace	http://gabrmn.uab.es/jmrui2xml_sv
properties	minOcc 0<br>maxOcc 1<br>content complex
children	**sv:NrPoints** **sv:MinPPM** **sv:MaxPPM**
source	<xs:element name="NumberOfPoints" minOccurs="0"><br><xs:complexType><br><xs:sequence><br><xs:element name="NrPoints" type="xs:integer"/><br><xs:element name="MinPPM" type="xs:double"/><br><xs:element name="MaxPPM" type="xs:double"/><br></xs:sequence><br></xs:complexType><br></xs:element>

element **DATASET/Preprocessing/NumberOfPoints/NrPoints**

diagram	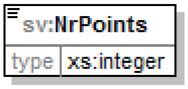
namespace	http://gabrmn.uab.es/jmrui2xml_sv
type	**xs:integer**
properties	content simple
source	<xs:element name="NrPoints" type="xs:integer"/>

element **DATASET/Preprocessing/NumberOfPoints/MinPPM**

diagram	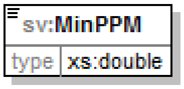
namespace	http://gabrmn.uab.es/jmrui2xml_sv
type	**xs:double**
properties	content simple
source	<xs:element name="MinPPM" type="xs:double"/>

element **DATASET/Preprocessing/NumberOfPoints/MaxPPM**

diagram	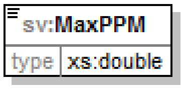
namespace	http://gabrmn.uab.es/jmrui2xml_sv
type	**xs:double**
properties	content simple
source	<xs:element name="MaxPPM" type="xs:double"/>

element **DATASET/Preprocessing/SetToUnitLength**

diagram	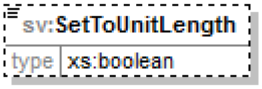
namespace	http://gabrmn.uab.es/jmrui2xml_sv
type	**xs:boolean**
properties	minOcc 0<br>maxOcc 1<br>content simple
source	<xs:element name="SetToUnitLength" type="xs:boolean" minOccurs="0"/>

element **DATASET/Preprocessing/AlignmentCorrection**

diagram

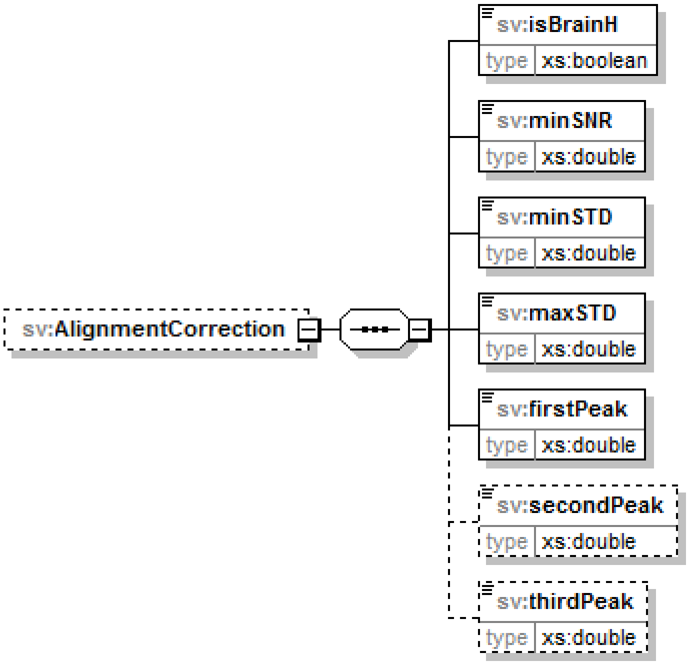

namespace: http://gabrmn.uab.es/jmrui2xml_sv

properties:

minOcc	0
maxOcc	1
content	complex

children: **sv:isBrainH sv:minSNR sv:minSTD sv:maxSTD sv:firstPeak sv:secondPeak sv:thirdPeak**

source

```
<xs:element name="AlignmentCorrection" minOccurs="0">
  <xs:complexType>
    <xs:sequence>
      <xs:element name="isBrainH" type="xs:boolean"/>
      <xs:element name="minSNR" type="xs:double"/>
      <xs:element name="minSTD" type="xs:double"/>
      <xs:element name="maxSTD" type="xs:double"/>
      <xs:element name="firstPeak" type="xs:double"/>
      <xs:element name="secondPeak" type="xs:double" minOccurs="0"/>
      <xs:element name="thirdPeak" type="xs:double" minOccurs="0"/>
    </xs:sequence>
  </xs:complexType>
</xs:element>
```

element **DATASET/Preprocessing/AlignmentCorrection/isBrainH**

diagram

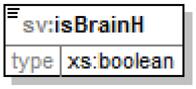

namespace: http://gabrmn.uab.es/jmrui2xml_sv

type	**xs:boolean**
properties	content simple
source	<xs:element name="isBrainH" type="xs:boolean"/>

element **DATASET/Preprocessing/AlignmentCorrection/minSNR**

diagram	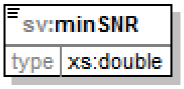
namespace	http://gabrmn.uab.es/jmrui2xml_sv
type	**xs:double**
properties	content simple
source	<xs:element name="minSNR" type="xs:double"/>

element **DATASET/Preprocessing/AlignmentCorrection/minSTD**

diagram	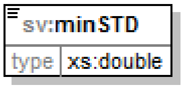
namespace	http://gabrmn.uab.es/jmrui2xml_sv
type	**xs:double**
properties	content simple
source	<xs:element name="minSTD" type="xs:double"/>

element **DATASET/Preprocessing/AlignmentCorrection/maxSTD**

diagram	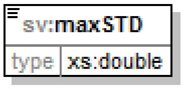
namespace	http://gabrmn.uab.es/jmrui2xml_sv
type	**xs:double**
properties	content simple
source	<xs:element name="maxSTD" type="xs:double"/>

element **DATASET/Preprocessing/AlignmentCorrection/firstPeak**

diagram	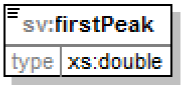
namespace	http://gabrmn.uab.es/jmrui2xml_sv

type	**xs:double**
properties	content simple
source	<xs:element name="firstPeak" type="xs:double"/>

element **DATASET/Preprocessing/AlignmentCorrection/secondPeak**

diagram	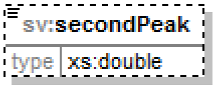
namespace	http://gabrmn.uab.es/jmrui2xml_sv
type	**xs:double**
properties	minOcc 0<br>maxOcc 1<br>content simple
source	<xs:element name="secondPeak" type="xs:double" minOccurs="0"/>

element **DATASET/Preprocessing/AlignmentCorrection/thirdPeak**

diagram	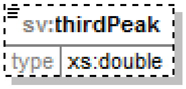
namespace	http://gabrmn.uab.es/jmrui2xml_sv
type	**xs:double**
properties	minOcc 0<br>maxOcc 1<br>content simple
source	<xs:element name="thirdPeak" type="xs:double" minOccurs="0"/>

element **DATASET/Preprocessing/AdditionalInformation**

diagram	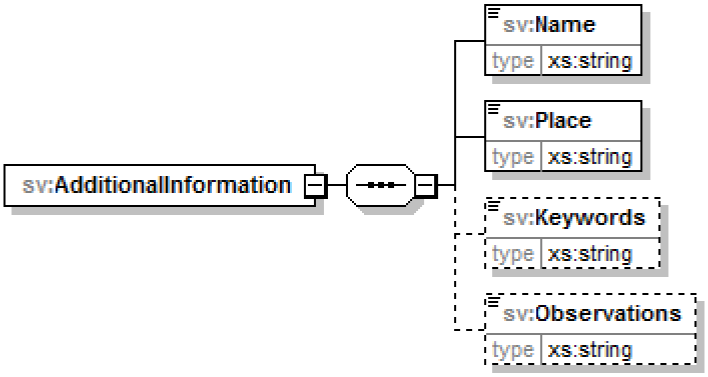
namespace	http://gabrmn.uab.es/jmrui2xml_sv
properties	content complex

children	sv:Name sv:Place sv:Keywords sv:Observations
source	<xs:element name="AdditionalInformation"><br><xs:complexType><br><xs:sequence><br><xs:element name="Name" type="xs:string"/><br><xs:element name="Place" type="xs:string"/><br><xs:element name="Keywords" type="xs:string" minOccurs="0"/><br><xs:element name="Observations" type="xs:string" minOccurs="0"/><br></xs:sequence><br></xs:complexType><br></xs:element>

element **DATASET/Preprocessing/AdditionalInformation/Name**

diagram	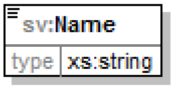
namespace	http://gabrmn.uab.es/jmrui2xml_sv
type	**xs:string**
properties	content simple
source	<xs:element name="Name" type="xs:string"/>

element **DATASET/Preprocessing/AdditionalInformation/Place**

diagram	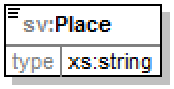
namespace	http://gabrmn.uab.es/jmrui2xml_sv
type	**xs:string**
properties	content simple
source	<xs:element name="Place" type="xs:string"/>

element **DATASET/Preprocessing/AdditionalInformation/Keywords**

diagram	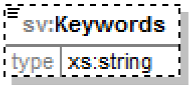
namespace	http://gabrmn.uab.es/jmrui2xml_sv
type	**xs:string**
properties	minOcc 0<br>maxOcc 1<br>content simple
source	<xs:element name="Keywords" type="xs:string" minOccurs="0"/>

element **DATASET/Preprocessing/AdditionalInformation/Observations**

diagram	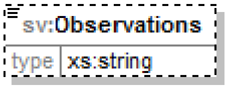
namespace	http://gabrmn.uab.es/jmrui2xml_sv
type	**xs:string**
properties	minOcc 0<br>maxOcc 1<br>content simple
source	<xs:element name="Observations" type="xs:string" minOccurs="0"/>

element **DATASET/Voxel**

diagram	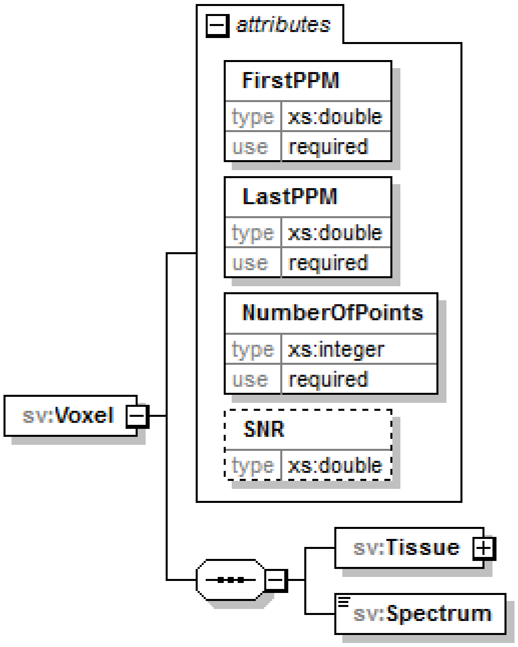
namespace	http://gabrmn.uab.es/jmrui2xml_sv
properties	content complex
children	**sv:Tissue** **sv:Spectrum**
attributes	Name / Type / Use / Default / Fixed / Annotation<br>FirstPPM / **xs:double** / required<br>LastPPM / **xs:double** / required<br>NumberOfPoints / **xs:integer** / required<br>SNR / **xs:double**
source	<xs:element name="Voxel"><br><xs:complexType><br><xs:sequence><br><xs:element name="Tissue">

```
        <xs:complexType>
          <xs:attribute name="Type" type="xs:string"/>
        </xs:complexType>
      </xs:element>
      <xs:element name="Spectrum"/>
    </xs:sequence>
    <xs:attribute name="FirstPPM" type="xs:double" use="required"/>
    <xs:attribute name="LastPPM" type="xs:double" use="required"/>
    <xs:attribute name="NumberOfPoints" type="xs:integer" use="required"/>
    <xs:attribute name="SNR" type="xs:double"/>
  </xs:complexType>
</xs:element>
```

attribute **DATASET/Voxel/@FirstPPM**

type	**xs:double**
properties	use required
source	<xs:attribute name="FirstPPM" type="xs:double" use="required"/>

attribute **DATASET/Voxel/@LastPPM**

type	**xs:double**
properties	use required
source	<xs:attribute name="LastPPM" type="xs:double" use="required"/>

attribute **DATASET/Voxel/@NumberOfPoints**

type	**xs:integer**
properties	use required
source	<xs:attribute name="NumberOfPoints" type="xs:integer" use="required"/>

attribute **DATASET/Voxel/@SNR**

type	**xs:double**
source	<xs:attribute name="SNR" type="xs:double"/>

element **DATASET/Voxel/Tissue**

diagram	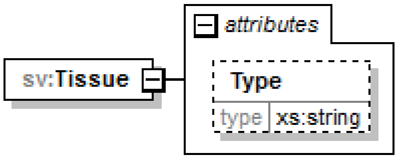
namespace	http://gabrmn.uab.es/jmrui2xml_sv

properties	content complex
attributes	Name Type Use Default Fixed Annotation<br>Type **xs:string**
source	<xs:element name="Tissue"><br><xs:complexType><br><xs:attribute name="Type" type="xs:string"/><br></xs:complexType><br></xs:element>

attribute **DATASET/Voxel/Tissue/@Type**

type	**xs:string**
source	<xs:attribute name="Type" type="xs:string"/>

element **DATASET/Voxel/Spectrum**

diagram	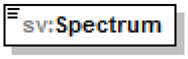
namespace	http://gabrmn.uab.es/jmrui2xml_sv
source	<xs:element name="Spectrum"/>

element **DATASET/Grid**

diagram	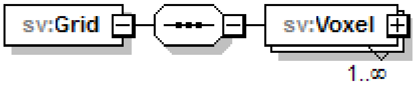
namespace	http://gabrmn.uab.es/jmrui2xml_sv
properties	content complex
children	**sv:Voxel**
source	<xs:element name="Grid"><br><xs:complexType><br><xs:sequence><br><xs:element name="Voxel" maxOccurs="unbounded"><br><xs:complexType><br><xs:sequence><br><xs:element name="Tissue"><br><xs:complexType><br><xs:attribute name="Type" type="xs:string"/><br></xs:complexType><br></xs:element><br><xs:element name="Spectrum"/><br></xs:sequence><br><xs:attribute name="FirstPPM" type="xs:double" use="required"/><br><xs:attribute name="LastPPM" type="xs:double" use="required"/><br><xs:attribute name="NumberOfPoints" type="xs:integer" use="required"/><br><xs:attribute name="Xaxis" type="xs:integer" use="required"/>

```
      <xs:attribute name="Yaxis" type="xs:integer" use="required"/>
      <xs:attribute name="Zaxis" type="xs:integer" use="required"/>
      <xs:attribute name="SNR" type="xs:double"/>
     </xs:complexType>
    </xs:element>
   </xs:sequence>
  </xs:complexType>
</xs:element>
```

element **DATASET/Grid/Voxel**

<table>
<tr><td>diagram</td><td>
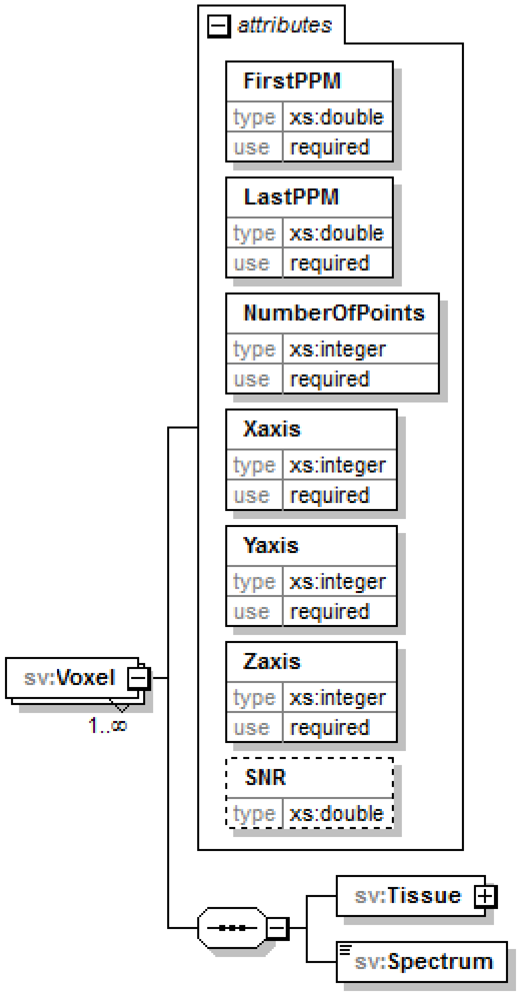

</td></tr>
<tr><td>namespace</td><td>http://gabrmn.uab.es/jmrui2xml_sv</td></tr>
<tr><td>properties</td><td>minOcc 1
maxOcc unbounded
content complex</td></tr>
<tr><td>children</td><td>sv:Tissue sv:Spectrum</td></tr>
<tr><td>attributes</td><td>
<table>
<tr><td>Name</td><td>Type</td><td>Use</td><td>Default</td><td>Fixed</td><td>Annotation</td></tr>
<tr><td>FirstPPM</td><td>xs:double</td><td>required</td><td></td><td></td><td></td></tr>
<tr><td>LastPPM</td><td>xs:double</td><td>required</td><td></td><td></td><td></td></tr>
</table>
</td></tr>
</table>

	NumberOfPoints	xs:integer	required
	Xaxis	xs:integer	required
	Yaxis	xs:integer	required
	Zaxis	xs:integer	required
	SNR	xs:double	

source

```
<xs:element name="Voxel" maxOccurs="unbounded">
  <xs:complexType>
    <xs:sequence>
      <xs:element name="Tissue">
        <xs:complexType>
          <xs:attribute name="Type" type="xs:string"/>
        </xs:complexType>
      </xs:element>
      <xs:element name="Spectrum"/>
    </xs:sequence>
    <xs:attribute name="FirstPPM" type="xs:double" use="required"/>
    <xs:attribute name="LastPPM" type="xs:double" use="required"/>
    <xs:attribute name="NumberOfPoints" type="xs:integer" use="required"/>
    <xs:attribute name="Xaxis" type="xs:integer" use="required"/>
    <xs:attribute name="Yaxis" type="xs:integer" use="required"/>
    <xs:attribute name="Zaxis" type="xs:integer" use="required"/>
    <xs:attribute name="SNR" type="xs:double"/>
  </xs:complexType>
</xs:element>
```

attribute **DATASET/Grid/Voxel/@FirstPPM**

type	**xs:double**
properties	use required
source	`<xs:attribute name="FirstPPM" type="xs:double" use="required"/>`

attribute **DATASET/Grid/Voxel/@LastPPM**

type	**xs:double**
properties	use required
source	`<xs:attribute name="LastPPM" type="xs:double" use="required"/>`

attribute **DATASET/Grid/Voxel/@NumberOfPoints**

type	**xs:integer**
properties	use required
source	`<xs:attribute name="NumberOfPoints" type="xs:integer" use="required"/>`

attribute **DATASET/Grid/Voxel/@Xaxis**

type	**xs:integer**
properties	use required
source	<xs:attribute name="Xaxis" type="xs:integer" use="required"/>

attribute **DATASET/Grid/Voxel/@Yaxis**

type	**xs:integer**
properties	use required
source	<xs:attribute name="Yaxis" type="xs:integer" use="required"/>

attribute **DATASET/Grid/Voxel/@Zaxis**

type	**xs:integer**
properties	use required
source	<xs:attribute name="Zaxis" type="xs:integer" use="required"/>

attribute **DATASET/Grid/Voxel/@SNR**

type	**xs:double**
source	<xs:attribute name="SNR" type="xs:double"/>

element **DATASET/Grid/Voxel/Tissue**

diagram	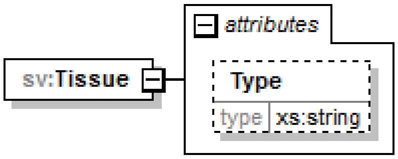
namespace	http://gabrmn.uab.es/jmrui2xml_sv
properties	content complex
attributes	Name: Type; Type: **xs:string**; Use; Default; Fixed; Annotation
source	<xs:element name="Tissue"><br><xs:complexType><br><xs:attribute name="Type" type="xs:string"/><br></xs:complexType><br></xs:element>

attribute **DATASET/Grid/Voxel/Tissue/@Type**

type	**xs:string**

source	<xs:attribute name="Type" type="xs:string"/>

element **DATASET/Grid/Voxel/Spectrum**

diagram	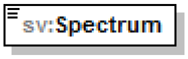
namespace	http://gabrmn.uab.es/jmrui2xml_sv
source	<xs:element name="Spectrum"/>
